# Simple Remedies

**Published:** 1883-10

**Authors:** 


### SIMPLE REMEDIES.

HALF a teaspoonful of common table salt dissolved in a little cold water, and drank, will generally relieve "HEART-BURN" or DYSPEPSIA. If taken every morning before breakfast, increasing the quantity gradually to a teaspoonful of salt and a tumbler of water, it will in a few days cure any ordinary case of DYSPEPSIA, if, at the same time due attention is paid to the diet. There is no better remedy than the above for CONSTIPATION. As a gargle for SORE THROAT it is equal to chlorate of potash, and it is entirely safe. It may be used as often as desired, and if a little is swallowed each time it will have a beneficial effect on the THROAT by cleansing it and by allaying the irritation. In doses of one to four teaspoonfuls in half-pint to a pint of tepid water, it acts promptly as an EMETIC; and in cases of poisoning is always at hand. It is an excellent remedy for BITES and STINGS of insects. It is a valuable astringent in HEMORRHAGES, particularly for bleeding after the extraction of teeth. It has both cleansing and healing properties, and is therefore a most excellent application for superficial ULCERATIONS. Try dry sulphur for canker in mouth or ulcerated throat.

MUSTARD is another valuabie remedy. No family should be without it. A teaspoonful of white mustard seed taken occasionly is a good remedy for heart-burn or dyspepsia. Two or three teaspoonfuls of ground mustard stirred into half pint of water acts as an emetic very promptly, and is milder and easier to take than salt and water. Equal parts of ground mustard and flour or meal, made into a paste with warm water, and spread on a thin piece of muslin with another piece of muslin laid over it, forms the often indispensable "mustard plaster." It is almost a spcific for COLIC when applied for a few minutes over the "pit of the stomach." For all INTERNAL PAINS and CONGESTIONS, there is no remedy of more general utility. It acts as a counterirritant, by drawing the blood to the surface; hence in severe cases of croup a small mustard plaster should be applied to the back of the child's neck. The same treatment will relieve almost any case of headache. A mustard plaster should be moved about over the spot to be acted upon, for if left too long in one place it is liable to blister. A mustard plaster acts well when at considerable distance from the affected part. An excellent substitute for mustard plaster is what is known as "Mustard Leaves." They come a dozen in a box and are about four or five inches in size; they are perfectly dry and will keep for a long time. For use, it is only necessary to dip one end in a dish of water for a minute and then apply it, between cloths or thin paper.

COMMON BAKING SODA is the best of all remedies in cases of scalds and burns. It may be used on the surface of the burned place, either dry or wet. When applied promptly, the sense of relief is magical. It seems to withdraw the heat and with it the pain, and the healing process soon commences. It is an excellent application for eruptions caused by poisonous ivy or other poisonous plants, as also for bites and stings of insects. Owing to colds, over-fatigue, anxiety and various other causes, the urine is often scanty, highly colored and more or less loaded with phosphates, which settle to the bottom of the vessel on cooling. As much soda as can be dipped up with a ten-cent piece, dissolved in half a glass of cold water and drank every three hours, will soon remedy the trouble and cause relief to the oppression that always exists from interruption of the natural flow of urine. This treatment should not be continued for more than twenty-four hours. Drinking of hot water as recommended in another article is recommended for this trouble. As a gargle for sore throat, a teaspoonful of salt, a tablespoonful of vinegar and as much water, with a sprinkling of cayenne pepper, and used as a gargle, usually affords relief. We have no more space to devote to this subject now; but it is one of universal interest, and we shall continue it. We shall endeavor to show that most of the diseases and accidents that are constantly occurring could be remedied or avoided by resorting to such remedies and appliances as are to be found in every home.

